# Performance of QuantiFERON-TB Gold In-Tube (QFTGIT) for the diagnosis of *Mycobacterium tuberculosis (Mtb) *infection in Afar Pastoralists, Ethiopia

**DOI:** 10.1186/1471-2334-10-354

**Published:** 2010-12-17

**Authors:** Mengistu Legesse, Gobena Ameni, Gezahegne Mamo, Girmay Medhin, Gunnar Bjune, Fekadu Abebe

**Affiliations:** 1 Aklilu Lemma Institute of Pathobiology, Addis Ababa University, Addis Ababa, Ethiopia; 2 Faculty of Veterinary Medicine, Addis Ababa University, Bishofituu, Ethiopia; 3 Department of General Practice and Community Medicine, Institute for Health and Society, University of Oslo, Oslo, Norway

## Abstract

**Background:**

Currently, T-cell based gamma interferon (IFNγ) release assays (IGRAs) are acknowledged as the best methods available for the screening of latent tuberculosis infection (LTBI) and also as aid for the diagnosis of active tuberculosis (TB). To our information, the performance of these diagnostic tests has not been evaluated in Ethiopia. Therefore, the intent of this study was to evaluate the performance of QuantiFERON-TB Gold In-Tube (QFTGIT) in patients clinically suspected of active pulmonary TB (PTB) as well as in healthy subjects prior to its utilization for the epidemiological study of active TB and LTBI in Afar pastoralists.

**Methods:**

The sensitivity of QFTGIT was evaluated in 140 subjects who were clinically suspected of PTB using the cut-off value recommended by the manufacturer (≥ 0.35 IU/ml) and disease-specific cut-off value. Sputum culture result was used as a gold standard. The specificity of the test was evaluated both in patients and in 55 tuberculin skin test (TST) negative healthy subjects.

**Results:**

Out of the 140 study participants, 37 (26.4%) were positive for active PTB by culture. Out of the 37 subjects who had positive results by culture, 6 individuals were HIV-seropositive. Out of the 103 subjects who were negative by culture, 6 subjects had indeterminate results and 21 were HIV-seropositive. The performance of the test was assessed using data from 107 (31 culture positive and 76 culture negative) individuals who were clinically suspected of PTB and HIV-seronegatives. Using the manufacturer recommended cut-off value, the sensitivity of the test was 64.5% (20/31), while its specificity was 36.8% (28/76). The sensitivity of the test was increased to 77.4%, while the specificity was reduced to 23.7% using a cut-off value ≥ 0.1 IU/ml of IFNγ as disease-specific cut-off value. In TST negative healthy subjects, the specificity of the test was 58.2%.

**Conclusion:**

Our findings revealed a low sensitivity of QFTGIT in the diagnosis of *Mycobacterium tuberculosis (Mtb) *infection in the present study area using the cut-off value recommended by the manufacturer. Nevertheless, the sensitivity increased from 64.5% to 77.4% by lowering the cut-off value recommended by the manufacturer to ≥ 0.1 IU/ml of IFNγ level. Hence, it is of practical importance to evaluate the performance of QFTGIT in population under different settings prior to its application either for the diagnosis of active TB or LTBI.

## Background

Approximately one third of the world's population is harbouring latent tuberculosis infection (LTBI) though most of the infected individuals do not develop active form of the disease [1]. Factors like HIV/AIDS and undernutrition that can affect the host immune response are substantially contributing to the re-activation of LTBI [2,3]. In the era of HIV/AIDS pandemic, there is a fear that over 200 million active tuberculosis (TB) cases and 35 million deaths might occur globally by 2020, if TB control is not increased [4]. Hence, screening and treating individuals harbouring LTBI may minimize the risk of subsequent development to active TB, especially in individuals at high risk of progression though this strategy is less applicable in areas where the disease is endemic, because of factors like high prevalence of LTBI, HIV/AIDS, malnutrition as well as the high cost of IGRAs.

Tuberculin skin test (TST), which has been used for the diagnosis of LTBI for a century [5], is relatively inexpensive and does not require laboratory facilities to perform. Nevertheless, its limitations like high rate of false-positive results in bacille Calmette-Guérin (BCG) vaccinated or in atypical environmental mycobacterium infected individuals as well as high rate of false-negative results among immunosuppressed individuals made difficult its utilization for the screening of LTBI, especially in regions where BCG vaccination and HIV/AIDS are common [6,7].

Alternative diagnostic methods to the TST, T-cell based gamma interferon (IFNγ) release assays (IGRAs) have been developed and approved mainly for the diagnosis of LTBI and also as aid for the diagnosis of active TB [8]. Currently, there are three (T-SPOT.TB, QntiFERON-TB Gold and QFTGIT) commercial available kits of IGRAs. T-SPOT.TB and QuantiFERON TB Gold assays use Early Secretory Antigenic Target (ESAT)-6 and Culture Filtrate Protein (CFP)-10 as specific antigens. Whereas, QFTGIT incorporates three *Mycobacterium tuberculosis (Mtb) *specific antigens (ESAT-6, CFP-10 and TB7.7).

IGRAs have been widely evaluated in active TB patients for the diagnosis of *Mtb *infection and various levels of sensitivities and specificities have been reported [9-16]. However, most of the information on the performance of tests has been reported from the developed countries, where the prevalence of TB is moderate or low. Thus, further information is needed on the performance of IGRAs from developing countries where TB is endemic as well as other factors such as helminthic infections, HIV/AIDS and undernutrition are prevalent [17,18]. On top of these, IGRAs have been found to be affected by factors like ethnic background and immune status of the population being tested [19,20]. The importance of revising the current recommended cut-off values of the tests based on evidence from various epidemiological and population context is also becoming an important issue [21,22]. Therefore, it is of practical importance to evaluate IGRAs in countries and population under different settings prior to its application for routine screening of active TB or epidemiological study of LTBI.

According to the World Health Organization (WHO) estimate, Ethiopia is ranked 7^th ^among the 22 countries with high-burden of TB in the world, while it stands second in Africa [3]. Nevertheless, to the best of our information, there is no published work that has evaluated the performance of QFTGIT for the diagnosis of *Mtb *infection in Ethiopia. Thus, as part of a large study on TB in Afar pastoral communities and their livestock, we assessed the performance of QFTGIT in subjects clinically suspected of PTB and apparently healthy individuals, using the cut-off value recommended by the manufacturer and disease-specific cut-off value.

## Methods

### Study Sites and Subjects

The study was conducted at Dubti Referral Hospital (DRH) and Awash Health Centre (AHC) in Afar region, North-East Ethiopia between December 2008 and March 2009. Dubti Hospital is a referral hospital for the region and it is located in Lower Awash valley, approximately at 574 km to the North-East of Addis Ababa. Awash Health centre is located in the Middle Awash valley at 220 km to the East of Addis Ababa. The majorities (80%) of the Afar population are pastoralists and they are distinct ethnic group [23]. TB is one of the major public health problems in Afar and the region is ranked 2^nd ^in Ethiopia with a notification rate between 146-260 per 100, 000 population [3,24].

Patients who visited outpatient department (OPD) of the two health facilities and who met the inclusion criteria were invited to participate in the study. Patients were eligible if they were clinically suspected of active PTB by physician, 18 years or above, volunteer to provide blood and sputum samples, volunteer to be tested for HIV infection and volunteer to provide written informed consent. Severely sick patients (include those patients who could not able to walk, sit, speak or give informed consent), anaemic patients and pregnant women were excluded from the study. Patients with known history of immunosuppressive therapies and who were on anti-TB treatment were also excluded from the study. Fifty five adult healthy pastoralists were also recruited in the study as control group. The inclusion criteria for the control group were: age between 18 and 30 years, TST negativity, absence of history of contact with TB patients, absence of previous history of TB, absence of symptoms of active TB, voluntariness to provide blood sample as well as to be tested for HIV infection and willingness to provide written informed consent.

Socio-demographic data like gender, age, ethnicity and occupation, as well as information on clinical/radiological findings and co-morbidities with other diseases were recorded by physician during clinical investigation. All study participants were also interviewed for previous history of TB treatment, contact with TB patients and BCG scar by physician. The study protocol was approved by the Ethical Clearance Committee of the Aklilu Lemma Institute of Pathobiology (ALIPB), Addis Ababa University and the Regional Committee for Medical Research Ethics of Southern Norway. Upon recruitment, the aim of the study was described to the study participants and written informed consent was obtained from each of the study participant.

### Sputum Collection and Examination

Three sputum specimens (spot-morning-spot), were obtained from each study participant, who was clinically suspected of PTB by a physician as previously recommended [25]. Briefly, the first sputum specimen was collected in the health facility (spot specimen) on the first day the patient visited the health facility. The second was an early morning sputum specimen (morning specimen), which was collected at home on the second day and delivered on the same day to the laboratory. The third sputum specimen was collected in the health facility (spot specimen) on the second day after delivering the morning specimen. Smear was prepared from a portion of each specimen, processed by the Ziehl-Neelsen (ZN) staining technique and microscopically examined for acid-fast bacillus (AFB) as previously described [26]. The remaining specimen was stored at 4°C until transported to the ALIPB Microbiology laboratory under cold condition. Upon reaching the laboratory, specimens were pooled together, decontaminated for 15 minutes with an equal volume of 4% NaOH and centrifuged at 3000 revolution per minute (rpm) for 15 minutes. The supernatant was decanted while the sediment was neutralized with 1% (0.1N) HCl using phenol red as an indicator. Neutralization was achieved when the colour of the solution was changed from purple to yellow. Then, 0.1 ml of the pellet was inoculated onto Lowenstein-Jensen medium containing pyruvate or glycerol and incubated for 10 weeks at 37°C [27]. Cultures were followed weekly for the growth of mycobaterial colonies and positivity for AFB was confirmed by microscopy.

### QuantiFERON-TB Gold In-Tube Assay

The QFTGIT was performed according to the manufacturer's instructions (QuantiFERON-TB Gold In-Tube, Cellestis Ltd., Carnegie, Australia). Briefly, on the day of sputum collection, 1 ml venous blood sample was collected from each individual directly into three tubes (TB-specific antigens, mitogen and nil tubes). The samples were incubated for 24 hours at 37°C in the laboratory of each health facility and then centrifuged at 3000 × rcf (relative centrifugal force) for 10 minutes. Plasma was collected and stored at 4°C until IFNγ assay was performed using ELISA. The optical density (OD) of each test was read using a 450 nm filter with a 620 nm reference filter using ELISA plate reader, and the results were interpreted as positive, negative or indeterminate using QFTGIT analysis software developed by the company (QFTGIT, Cellestis Ltd., Carnegie, Australia).

### Determination of Specificity of the Test

The specificity of the test was assessed using blood sample collected from 55 apparently healthy adult pastoralists who were negative for LTBI by TST. They were also seronegative for HIV infection.

### Tuberculin Skin Testing in Healthy Control Subjects

Immediately following blood collection from the right hand, 0.1 ml (2T.U/0.1 ml) Tuberculin PPD RT23 (Statens Serum Institute, Copenhagen, Denmark) was administrated intradermally in the middle third of the left forearm by well experienced nurse. The diameter induration transverse to the long axis of the forearm was measured between 48-to-72 hours using flexible plastic ruler. Diameter of skin induration < 6 mm was considered as negative for the test as recommended by the manufacturer.

### Estimation of Disease-specific Cut-off Value

A receiver operator characteristic (ROC) curve analysis was used to estimate disease-specific cut-off value for QFTGIT using data of patients clinically suspected of PTB and healthy subjects as previously described [11,22].

### Stool and Urine Samples Collection and Examination

Stool and urine samples were collected from the study participants, processed by the Kato and filtration techniques, respectively. The stool samples were microscopically examined for ova of *Schistosoma mansoni *(*Sm*)/other intestinal helminths, while urine samples were microscopically examined for ova of *Schistosoma haematobium *(*Sh*) as previously described [28].

### Data Analysis

Data were entered into EpiData Software version 3.1 and analyzed using Stata version 8 (StataCorp LP). Means were compared using the Student's t test for two independent samples. Sensitivity and specificity of QFTGIT were estimated using (a) the cut-off value recommended by the manufacturer (≥0.35, IU/ml) and (b) ROC using the data generated from the current study (disease-specific cut-off value). Sensitivity was calculated by dividing the number of patients who were positive by culture and QFTGIT by the number of true positives (i.e. culture positive). In the case of suspected patients of PTB, the specificity of the test was evaluated by dividing the number of individuals with a negative test both by the QFTGIT and culture by the number of total negative (i.e. culture negative). In the case of healthy subjects, the specificity of the test was evaluated by dividing the number of individuals with a negative test both by the QFTGIT and TST by the number of total negatives by the TST. The effect of socio-demographic variables on the diagnostic performance of QFTGIT was evaluated using univariable and multivariable logistic regression. P value less than 0.05 was considered significant in all analysis.

## Results

### Baseline Characteristics of Study Patients

A total of 167 (132 from DRH and 35 from AHC) study participants who were clinically suspected of PTB were requested to be involved in the study. Out of these subjects, 27(16.2%) did not volunteer to provide complete data. Hence, data were analyzed for 140 subjects (108 from DRH and 32 from AHC) whose age range was between 18 and 70 years (mean age 34.2 years). More than half of the study participants were males (54.3%), belong to Afar ethnic group (72.9%) and pastoralists by occupation (56.4%).

Out of 140 subjects who were tested for HIV infection, 27 (19.3%) were seropositive. Fifty four (38.6%) subjects had chest radiograph results of whom 28 (51.9%) had suggestive results for PTB. The rest, 86 (61.4%) subjects had no results on chest radiograph as radiograph examination for these individuals was not requested by the physician. Thirty four (24.3%) and 6 (4.3%) subjects reported previous history of contact with TB patients and previous history of TB treatment, respectively. Twenty eight (20.0%) subjects had BCG scar.

### Bacteriological Assessment

Out of the 140 study participants, 37 (26.4%) and 12 (8.6%) were positive for active PTB by culture and direct AFB smear microscopy, respectively. Of the 12 study participants who had positive results by direct AFB smear microscopy, 2 were negative by culture. Out of the 37 subjects who had positive results by culture, 6 individuals were HIV-seropositive. Of the 28 individuals who had positive results by chest radiography, 4 (14.3%) were positive by culture.

### IFNγ Assay Using Cut-off Value Recommended by the Manufacturer

Out of the 140 participants who were clinically suspected of PTB, 83 (59.3%) were positive for *Mtb *infection by QFTGIT using the cut-off value recommended by the manufacturer. Seven (5%) individuals (6 from DRH and 1 from AHC) had indeterminate results. Out of the 27 participants who were seropositive for HIV infection, 15 (55.6%), 11(40.7%), and 1(3.7%) had positive, negative and indeterminate results by QFTGIT, respectively. Out of the 37 culture confirmed PTB patients, 12 (32.4%) were negative by QFTGIT. Out of these 12 subjects, 1 was seropositive for HIV infection. Among 28 participants who had positive chest radiography for PTB, 18 (64.3%), 6 (21.4%) and 4 (14.3%) had positive, negative and indeterminate results by QFTGIT, respectively.

Results from logistic regression analysis showed that individuals in the age group between 30 to 44 years (24 out of 47, 51.1%) were more likely to have a negative QFTGIT results compared to those individuals who were younger than 30 years (16 out of 60, 26.7%) (Crude OR = 0.35; 95% CI, 0.16 to 0.78; p = 0.011, adjusted OR = 0.39; 95% CI, 0.16 to 0.98; p = 0.046). On the other hand, there was no significant association between the results of QFTGIT and participants' socio-demographic characteristics such as gender, ethnicity, BCG scar or occupation (Table 1).

**Table 1:** Association of study participants' socio-demographic characteristics with QFTGIT result

Characteristic	Crude OR(95%, CI)	Adjusted OR(95%, CI)
**Gender:**		
Female (n = 64)	Reference	Reference
Male (n = 76)	1.30 (0.65-2.62)	1.15 (0.55-2.4)
**Age (years):**		
18-29 (n = 60)	Reference	Reference
30-44 (n = 47)	0.35 (0.16-0.78)	0.39 (0.16-0.98)
45+ (n = 33)	0.84 (0.33-2.1)	0.79 (0.29-2.21)
**Ethnicity**		
Afar (n = 102)	Reference	Reference
Other (n = 38)	1.81 (0.79-4.12)	0.80 (0.21-3.07)
**Occupation **:		
Pastoralist (n = 79)	Reference	Reference
Other (n = 61)	1.63 (0.79-3.31)	1.49 (0.51-4.33)
**BCG scar present**		
Yes (n = 28)	Reference	Reference
No (n = 112)	2.37 (0.89-6.32)	1.45 (0.38-5.55)
**Previous TB treatment:**		
Yes (n = 6)	Reference	Reference
No (n = 134)	2.9 (0.33-25.38)	1.49 (0.12-17.17)
**Contact with TB patient:**		
Yes (n = 34)	Reference	Reference
No (n = 106)	1.75 (0.74-4.13)	1.71 (0.66-4.47)
**HIV status**:		
Positive (n = 27)	Reference	Reference
Negative (n = 113)	0.77 (0.32-1.81)	0.75 (0.28-2.02)

### Sensitivity and Specificity of QFTGIT in HIV-Seronegative study patients

Data from 27 participants who were seropositive for HIV infection was excluded from the analysis of the sensitivity and specificity of QFTGIT with the assumption that infection with HIV could affect either the bacteriological or QFTGIT results. Similarly, data from 6 individuals who had indeterminate results was also excluded from sensitivity/specificity analysis. Hence, the performance of the test was assessed using data from 107 (31 culture positive and 76 culture negative) individuals who were clinically suspected of PTB. Out of the 107 subjects, 20 (18.7%) were positive both by culture and QFTGIT, while 28 (26.2%) were negative by both tests (Table 2). Using the manufacturer recommended cut-off value, the sensitivity of the test was 64.5% (95% CI, 45.4 to 80.8), while its specificity was 36.8% (95% CI, 26.1 to 48.7). The positive and negative predicative values of the test were 29.4% (95% CI, 19.0 to 41.7) and 71.8% (95% CI, 55.1 to 85.0), respectively. Table 2 depicts the sensitivity and specificity of the QFTGIT at various cut-off values of the level of IFNγ.

**Table 2:** Diagnosis of Mtb infection by culture method and QFTGIT at various cut-off values of the level of INFγ (IU/ml) in HIV-seronegative study participants (n = 107)

		Culture method		Diagnostic performance	
**QFTGIT/cut-off value (IU/ml)**		**Number of positive**	**Number of negative**	**Sensitivity (%)**	**Specificity (%)**
≥ 0.35*	Number of positive	20	48	64.5	36.8
	Number of negative	11	28		
≥ 0.2	Number of positive	21	54	67.7	28.9
	Number of negative	10	22		
≥ 0.1	Number of positive	24	58	77.4	23.7
	Number of negative	7	18		

*Manufacturer's recommended cut-off value.

When the disease-specific cut-off value was estimated to be ≥ 0.2 IU/ml of IFNγ level, the sensitivity of the test increased from 64.5% to 67.7% (95% CI, 48.6 to 83.3), while the specificity was slightly decreased from 36.8% to 28.9% (95% CI, 19.1 to 40.5). Among 11 patients who were positive for PTB by culture, but negative by QFTGIT at the cut-off value ≥ 0.35 IU/ml, 1 was found to be positive at the cut-off value ≥ 0.2 IU/ml. Out of the 28 patients who were negatives by QFTGIT at the cut-off value ≥ 0.35 IU/ml, 6 (21.4%) were found to be positive using cut-off value ≥ 0.2 IU/ml. Among 32 healthy subjects who were negative by QFTGIT at cut-off value ≥ 0.35 IU/ml, 10 (31.3%) were found to be positive for LTBI using cut-off value ≥ 0.2 IU/ml of IFNγ level.

A cut-off value of ≥ 0.1 IU/ml of IFNγ level increased the sensitivity of the test from 64.5% to 77.4% (95% CI, 58.9 to 90.4), while the specificity decreased from 36.8% to 23.7% (95% CI, 14.7 to 34.8). Of those 11 patients who were positive for active PTB by culture but negative by QFTGIT, 4 (36.4%) were found to be positive by QFTGIT using cut-off value ≥ 0.1 IU/ml. Among 28 patients who were clinically suspected of PTB, but negative both by culture and QFTGIT at the cut-off value ≥ 0.35 IU/ml of IFNγ level, 10 (35.7%) were found to be positive using cut-off value ≥ 0.1 IU/ml. Similarly, of the 32 healthy subjects who were negative at the cut-off value ≥ 0.35 IU/ml, 13 (40.6%) were found to be positive for LTBI using cut-off value ≥ 0.1 IU/ml IFNγ level.

### Sensitivity and Specificity of QFTGIT in HIV-seropositive Study Patients

Out of the 27 HIV-seropositive subjects, 5 (18.5%) were positive both by culture and QFTGIT, whereas 10 (37.0%) subjects were negative by both tests (Table 3). All the 11 subjects who had negative results by QFTGIT, had < 0.1 IU/ml of IFNγ level. Hence, the sensitivity and specificity of QFTGIT were assessed at the manufacturer recommended cut-off value. Data from one subject who had indeterminate result was excluded from the analysis. The sensitivity and the specificity of the test were 83.3% and 50%, respectively.

**Table 3:** Diagnosis of Mtb infection by culture method and QFTGIT at cut-off value recommended by the manufacturer in HIV-seropositive study participants (n = 26)

		Culture method		Diagnostic performance	
**QFTGIT/cut-off value (IU/ml)**		**Number of positive**	**Number of negative**	**Sensitivity(%)**	**Specificity(%)**
≥ 0.35*	Number of positive	5	10	83.3	50.0
	Number of negative	1	10		

*Manufacturer's recommended cut-off value.

### Specificity of QFTGIT in Control Healthy Subjects

Out of 55 healthy adult pastoralists (14 females and 41 males, age range 18-30, mean age 23.6 years) who were negative by TST, 23 (41.8%) were diagnosed as positive for LTBI by QFTGIT. Hence, the specificity of QFTGIT was found to be 58.2% (95% CI, 44.1 to 71.3).

### Comparison of the Level of IFNγ in HIV-seronegative Study patients

**Figure 1:**
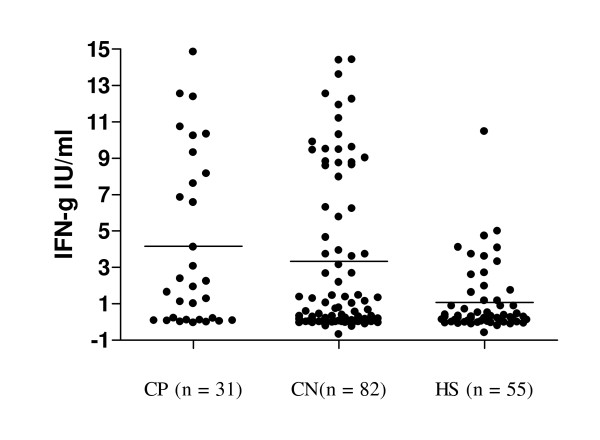
**Level of IFNγ induced by specific antigens in three groups of study participants**. Whole blood samples were collected from study participants suspected of pulmonary tuberculosis and apparently healthy subjects directly into tubes containing TB-specific antigens, mitogen and nil. The samples were incubated for 24 hours at 37°C, plasma was collected and IFNγ assay was performed using ELISA. The optical density (OD) of each test was read using a 450 nm filter with a 620 nm reference filter using ELISA plate reader, and the level of IFNγ (IU/ml) was interpreted using QuantiFERON-TB Gold In-Tube (QFTGIT) analysis software developed by the company (Cellestis Ltd., Carnegie, Australia). The subjects were categorized in to three groups based on sputum culture results as CP = sputum culture positive, CN = sputum culture negative and HS = healthy subjects and the mean levels of IFNγ IU/ml were compared for the three groups. Each dot represents the concentration of IFNγ IU/ml of each individual and the solid line shows the mean level of the IFNγ.

Figure 1 shows the mean levels of IFNγ induced by the specific antigens (ESAT-6, CFP-10 and TB7.7) in three sub groups (culture positive, culture negative and control healthy subjects) of the study participants. The results of one-way analysis of variance showed that the mean levels of IFNγ induced by the specific antigens in the three sub groups of the study participants were not equal (F = 8.18; df = 2, p = 0.0004). Further pair-wise analysis showed that the mean levels of IFNγ was not significantly different in study participants with culture negative (n = 82) and culture positive (n = 31) (3.33 ± 4.43 IU/ml vs 4.16 ± 4.72 IU/ml, p = 0.3850). Similarly, there was no statistically significant difference in the mean levels of IFNγ in participants who were positive both by culture and QFTGIT (n = 20) and those individuals who were negative by culture (n = 48), but diagnosed positive by QFTGIT (6.41 ± 4.49 IU/ml vs 5.69 ± 4.48 IU/ml, p = 0.5473). On the other hand, the mean level of IFNγ was significantly higher in study participants (n = 113) who were clinically suspected of PTB than that of the 55 healthy subjects (3.56 ± 4. 51 IU/ml vs 1.08 ± 1.90 IU/ml, p = 0.0002) (Figure 1). The mean level of IFNγ in those 68 study participants who were clinically suspected of PTB and diagnosed positive by QFTGIT was significantly higher than that of 23 healthy individuals who were positive for LTBI by QFTGIT (5.9 ± 4.46 IU/ml vs 2.46 ± 2.32 IU/ml, p = 0.0007). Similarly, a significant difference was found between mean levels of IFNγ of those study participants who were negative by culture, but positive by QFTGIT (n = 48) and those 23 healthy individuals who were positive for LTBI by QFTGIT (5.69 ± 4.48 IU/ml vs 2.46 ± 2.32 IU/ml, p = 0.0018).

**Figure 2:**
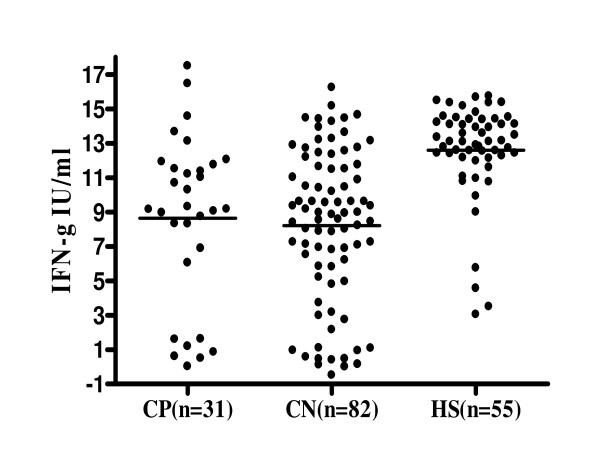
**Level of IFNγ induced by mitogen in three groups of study participants**.

Figure 2 shows the mean levels of IFNγ induced by the mitogen in the three sub groups of the study participants. The results of one-way analysis of variance showed that the mean levels of IFNγ induced by the mitogen in the three sub groups of the study participants were not equal (F = 20.51; df = 2, p < 0.001). Further pair-wise analysis showed that the mean levels of IFNγ induced by the mitogen were not significantly different in study participants with culture negative (n = 82) and culture positive (n = 31) (8.21 ± 4.44 IU/ml vs 8.63 ± 4.89 IU/ml, p = 0.6605). In contrast, the mean level of IFNγ induced by the mitogen was significantly higher in 55 control healthy subjects than in 113 study participants who were clinically suspected of PTB (12.6 ± 2.80 IU/ml vs 8.33 ± 4. 55 IU/ml, p < 0.001). The mean level of IFNγ induced by the mitogen was significantly higher in 55 control healthy subjects than that of 82 culture negative subjects (12.6 ± 2.80 IU/ml vs 8.21 ± 4.44 IU/ml, P < 0.001) and also than in 31 culture positive subjects (12.6 ± 2.80 IU/ml vs 8.63 ± 4.89 IU/ml, p < 0.001).

### Stool and Urine Examination

Out the total 140 subjects who were clinically suspected of PTB, 126 (90.0%) provided stool samples and 140 (100%) provided urine samples. In this group of study participants, only 2 (1.4%) individuals were positive for *Sh *infection. These 2 individuals were also positive by QFTGIT. Out of the 55 healthy individuals, 9 (16.4%) were positive for Sh infection, of whom 4 were positive for LTBI by QFTGIT.

## Discussion

In this study, we evaluated the diagnostic performance of QFTGIT in subjects clinically suspected of PTB using culture result as gold reference in area where the disease is highly prevalent [3,24]. Using the manufacturer recommended cut-off value; we found sensitivity of 64.5% and specificity of 36.8% of the test. The sensitivity of the test observed in the present study is much lower than sensitivity reported from TB endemic areas using the same test [9,29]. The discrepancy between the present study and these previous studies could be explained by multiple factors like population background and immune status, the clinical (disease) status and the socio-demographic characteristics of the study participants [14,19,20,30]. In the present study, 11 HIV-seronegative individuals who were positive for active PTB by culture were found to be negative by QFTGIT at cut-off value recommended by the manufacturer. Hence, the false negativity rate of the test was 35.5%, which is higher than the false negative rates reported for QFTGIT or other IGRAs by previous studies [9,14,20,31,32]. False negative results related to IGRAs have been associated with various factors such as impaired immune status, age of patients, the infecting *Mtb *strains, genetic background of the population or other chronic diseases [19,20,31-33]. Although QFTGIT was performed according to the manufacturer's instructions, factors like inadequate shaking of blood samples, incubation delay and power outages during incubation of blood samples are also inevitable assay-related problems which could be another possible explanation for the false-negative results of the test [34]. Hence, further studies are warranted to investigate factors contribute to false negative results of the QFTGIT in the present study area.

Study done on the evaluation of cut-off values of IGRAs in the diagnosis of *Mtb *infection in Turkey revealed that reducing the cut-off value to ≥ 0.1 IU/ml of IFNγ improved the sensitivity of the test by 13.8% [22]. Lee and his co-workers [11] also showed an improved sensitivity (from 70.1% to 86.2%) of the test by reducing the cut-off value to ≥ 0.13 IU/ml of IFNγ. Study in Zambian adults [32] also showed an increased sensitivity of the test from 74% to 82 by using a cut-off value ≥ 0.13 IU/ml of IFNγ. Study in Brazilian TB patients [32] also showed an increased sensitivity by 10% by lowering the cut-off value to 0.2 IU/ml of IFNγ. In the present study, reducing of the cut-off value to ≥ 0.1 IU/ml of IFNγ increased the sensitivity of QFTGIT from 64.5% to 77.4%. Thus, it is also reasonable to suppose that false negative results could occur because of the present high cut-off value of the test recommended by the manufacturer.

Studies showed that lowering of the cut-off value recommended by the manufacturer could decrease the specificity of the test based on the epidemiological status of the disease, though it improves sensitivity [11,22]. Some studies suggested the importance of reducing the present cut-off point recommended by the manufacturer for the diagnosis of active TB in areas where TB and HIV are prevalent regardless of its specificity [21,22,31]. In our study, out of those 11 patients who were positive for active PTB by culture, but negative by QFTGIT, 4 (36.4%) were found to be positive by QFTGIT using cut-off value ≥ 0.1 IU/ml. Similarly, among 28 patients who were clinically suspected of PTB, but negative by culture and QFTGIT at the cut-off value ≥ 0.35 IU/ml of IFNγ level, 10 (35.7%) were found to be positive using cut-off value ≥ 0.1 IU/ml. Logically, a test with high specificity is important in areas with a high prevalence of TB in order to minimize unnecessary treatment with anti-TB drugs. Nonetheless, taking into consideration the burden of TB in the present study area, we maintain the previous notion [21,22,31] that using low cut-off value of QFTGIT could aid for the screening of active TB in combination with clinical features though further studies on the immune profile of the Afar pastoralists are important.

Because of the lack of a gold standard test that accurately identifies subjects without LTBI, many investigators have evaluated only the sensitivity of the IGRAs either in culture proven or using combined results of culture and clinical findings as gold reference [14,20,35]. In the present study, we observed very low specificity of QFTGIT in patients clinically suspected of PTB compared to the specificity reported from elsewhere [9]. This poor specificity could be due to the high prevalence of TB in the present study area [3].

We also found a low specificity of QFTGIT in TST negative healthy subjects which is inconsistent with previous studies which showed high specificity rates of IGRAs in TST negative healthy individuals [10,11,32]. Among 55 healthy subjects who had skin induration < 5 mm, 23 (41.8%) subjects were found positive for LTBI by QFTGIT at the cut-off point recommended by the manufacturer. This could be explained by several factors such as: 1) False-negative results/low sensitivity of TST in subjects truly infected with *Mtb*, but un-reactive due to diminishing of the response over time [36]. 2) Undernutrition associated immunosuppression [37]. 3) The difference in the potential of the antigens (ESAT-6, CFP10) to stimulate specific IFNγ secreting effector T-cells [38]. 4) Although TST was performed by experienced nurse, it is impossible to exclude the effect of technical-related factors like errors in administration of the tuberculin or incorrect reading of the skin induration on the performance and interpretation of the results. Thus, further study is needed to elucidate factors hampered the sensitivity of TST, as compared to high sensitivity of the QFTGIT in the present study area.

In this study, we also observed a low positive predictive value of QFTGIT compared to reports from a low endemic setting [39]. This low positive predictive value of the test might be explained mainly by the high prevalence of LTBI in the present study area. Study showed that IGRAs are more sensitive in the diagnosis of patients clinically suspected of active TB than culture method [40]. In the present study, among 68 subjects who were positive by QFTGIT, 48 (70.6%) were found negative by culture method. Since culture method may not also absolutely classify all clinically suspected patients as patients with and without active TB, it is reasonable to presume that culture method could result in false negative which could affect both the sensitivity and the true positive predictive value of the test.

In the present study, QFTGIT revealed an overall positivity rate of 59.3% in patients clinically suspected of PTB. This positivity rate had no association with patients' baseline characteristics such as gender, ethnicity, occupation or HIV status. Study showed that HIV-infection increases the risk of indeterminate and false negative results by QFTGIT in active TB patients, whereas it decreases the sensitivity of the test, especially in patients with low CD4+ cell count [31]. In this study, indeterminate/false negative results or insensitivity of QFTGIT was not associated with HIV-seropositivity. This finding indicates that QFTGIT could help in the screening of LTBI as well as in the diagnosis of active TB in HIV-infected subjects though evidence on the status of CD4+ cells is crucial [41].

*In vitro *analysis of the responses CD4 T cells to either ESAT-6 or short-term culture filtrate (ST-CF) using other immunological assays indicated that *Mtb *infection induces high level of IFNγ in asymptomatic healthy subjects with LTBI compared to that of patients with active TB [30,42,43]. In this study, we found that the level of IFNγ induced by specific antigens in culture positive as well as in clinically suspected patients was higher than that of the control healthy subjects with LTBI. Previous studies also demonstrated that T-cell responses to specific-mycobacterium antigens measured using IGRAs is significantly higher in patients with active TB or clinically suspected cases than control healthy subjects with LTBI [10,11,35]. These findings necessitate further studies on the potential use of IGRAs for the diagnosis of active TB in combination with bacteriological and clinical features.

In this study, we could not involve a large number of study participants because of the high cost of QFTGIT. Hence, this small sample size of the study participants might limit the generalization of the findings according to the different subgroup of the study participants. Another limitation of this study is the incapability to provide a reliable data on the specificity of the QFTGIT because of the lack of gold standard for the diagnosis LTBI as well as the TB high-burden setting of the study area. Nevertheless, we believe that our study would provide important information on the performance of QFTGIT particularly in the present study setting where data is not available, and generally in high TB endemic settings.

## Conclusions

Our findings revealed a low sensitivity of QFTGIT in the diagnosis of *Mtb *infection in the present study population using the cut-off value recommended by the manufacturer. Nevertheless, the sensitivity increased from 64.5% to 77.4% by lowering the cut-off value recommended by the manufacturer to ≥ 0.1 IU/ml of IFNγ. Hence, it is of practical importance to evaluate the best disease-specific cut-off value that could improve the sensitivity of QFTGIT in population under different settings prior to its application for routine diagnosis of active TB or epidemiological study of LTBI.

## Competing interests

The authors declare that they have no competing interests.

## Authors' contributions

ML designed the study, participated in data collection, analysis and drafted the manuscript. GA, participated in study design, data collection, analysis and write-up. GM participated in study design, data collection and write-up. GMD, participated in study design, data analysis/interpretation and write-up. GB involved in study design and write-up manuscript. FA involved in study design, data analysis and write-up of the manuscript and critically revised the manuscript. All authors read and approved the final manuscript. ML is the guarantor of the paper.

## Pre-publication history

The pre-publication history for this paper can be accessed here:

http://www.biomedcentral.com/1471-2334/10/354/prepub
